# Impact of double moisture technique on throat pain and dysphagia among post-operative Indian patients: A randomized controlled trial

**DOI:** 10.6026/9732063002001989

**Published:** 2024-12-31

**Authors:** Venba Elangovan, Theranirajan Ethiraj, Shankar Shanmugam Rajendran, Anandhi Duraikannu, Divya Bharathi Jayaraman, Sudha Devadoss, Sundari Mani

**Affiliations:** 1 Department of Medical Surgical Nursing, College of Nursing, Madras Medical College, The TN Dr MGR Medical University, Chennai, Tamil Nadu, India; 2 Department of Paediatrics, Madras Medical College, The TN Dr MGR Medical University, Chennai, Tamil Nadu, India; 3 Department of Paediatric Nursing, College of Nursing, Madras Medical College, The TN Dr MGR Medical University, Chennai, Tamil Nadu, India; 4 Department of Medical Surgical Nursing, College of Nursing, Madras Medical College, The TN Dr MGR Medical University, Chennai, Tamil Nadu, India

**Keywords:** Double moisture technique, throat pain, dysphagia, endotracheal intubation

## Abstract

Post-operative pharyngeal discomfort and dysphagia are common issues that interfere with recovery, especially after surgeries involving general anesthesia and endotracheal intubation. A randomized controlled trial was conducted to evaluate the effectiveness of the double moisture technique in reducing sore throat and dysphagia in surgical recovery. Sixty patients were divided into experimental and control groups, with 30 patients each. The experimental group received steam inhalation and saline gargling, while the control group received routine treatment. Assessments were made on the second and third days post-intervention using standardized measures for throat pain and dysphagia. The results indicated a significant decrease in pain and dysphagia scores in the experimental group compared to pre-test values (F = 178.89, P ≤ 0.001; F = 213.76, P ≤ 0.001). The intervention group showed a 44.30% reduction in pain and a 21.58% reduction in dysphagia, while the control group had reductions of 20.60% and 9.66%, respectively. Demographic factors, such as age, comorbidities, BMI, activity level and occupation, also influenced the outcomes in the experimental group.

## Background:

Postoperative sore throat appears to be associated with airway manipulation and anesthesia. It has been linked to mucosal edema or dehydration, tracheal ischemia caused by the pressure and type of endotracheal tube cuffs, aggressive oropharyngeal suctioning, pathological changes, nerve damage and mucosal erosion caused by friction between delicate tissues and the size of the endotracheal tube. However, the specific cause remains uncertain [1]. Post-extubation dysphagia (PED) refers to difficulty swallowing after the removal of an endotracheal tube. This condition is relatively common, particularly in patients undergoing prolonged intubation or surgeries involving the neck or upper airway [2]. The causes of dysphagia include trauma to the airway, laryngeal edema, decreased sensation, muscle weakness and neuromuscular dysfunction. Clinical manifestations include coughing during or after swallowing, hoarseness, difficulty initiating a swallow, a sensation of food being stuck in the throat and drooling. Complications may include aspiration, pneumonia, malnutrition and prolonged hospital stays [3]. The method by which salt water gargling relieves inflammation and draws extra fluid out of swollen tissues is by producing a hypertonic environment in the throat, aids in the loosening of mucus and debris, facilitates the removal of bacteria and irritants from the throat and also offers momentary relief from pain and discomfort [4]. Steam inhalation enhances airway clearance by loosening secretions, improving circulation and soothing the mucous membrane, thereby reducing throat pain caused by endotracheal intubation [5, 6]. Therefore it is of interest to study the effectiveness of the double moisture technique on throat pain and dysphagia in post-operative patients.

## Statement of the problem:

The effectiveness of the double moisture technique on throat pain and dysphagia among postoperative patients in selected surgical wards at Tertiary Care Centre, Chennai.

## Objectives:

Our interest is to evaluate the level of dysphagia and throat aches in patients post-surgery on treatment and control groups before and after intervention. For this, initial pre-test data will be collated that would give a baseline assessment of throat pain and dysphagia. After the dual moisture method has been used, the post-assessment evaluations of the experimental group would then be conducted to detect changes in their symptoms against their counterparts of the control group. The developed method would then also be judged effectively by assessing the post-test results from the two groups. Therefore, it is of interest to establish the correlation between post-test throat discomfort and dysphagia levels with specific demographic and clinical variables within each cohort.

## Hypothesis:

H1: It is anticipated that there will be a noteworthy distinction in the post-test levels of dysphagia and throat pain between the experimental and control groups.

H2: A noteworthy correlation is expected to exist between the experimental group's selected demographic and clinical factors and the post-test levels of dysphagia and throat pain.

## Materials and Methods:

A randomized controlled study with a pre-test-post-test-only design was used to conduct a quantitative analysis among postoperative patients who had undergone a surgical procedure with endotracheal intubation in chosen surgical wards at the Institute of General Surgery, Rajiv Gandhi Government General Hospital, Chennai, after obtaining permission from Director and ahead of the Department. To establish feasibility, a pilot study was conducted among 10% of the calculated sample size. The researcher chose 60 postoperative patients using a simple random sampling technique and a lottery method and randomly split them into an experimental and control group (30 each). Post-operative patients with throat pain and dysphagia after extubation, within 1-3 postoperative days, initiated with oral feeds, consciousness and able to follow instructions, willing to participate and have given written consent were included in the study. Post-operative patients with a history of dysphagia due to any other comorbid conditions, post-operative complications, on oxygen and inotropic supports, less than 18 years of age were excluded.

Instruments used in the study include demographic and clinical information of the patients, a numeric scale to measure throat pain and the newly developed Eating Assessment Tool-10 (EAT-10) that measures dysphagia. Following the pre-test, the doubled moisture technique was given to the experimental group as the intervention for saltwater gargling (5 times gargling within 3-5 minutes) and steam inhalation (10 minutes) 2 times a day for 3 days. In contrast, routine nursing care was supplied to the control group. Using the same structured questionnaire, a post-test was administered on the 2nd and 3rd day after providing interventions. Data collected from the post-test was compiled and assessed using appropriate descriptive and inferential statistics. A two-independent samples t-test and an F-test for a one-way repeated measures ANOVA were used to determine the differences in outcomes between experimental and control groups. A paired t-test was used to compare differences in scores of the same group before and after testing. Lastly, a Chi-square test was deployed to check an association between two independent groups on the one hand and the relationship of post-test scores with myriad clinical and demographic attributes on the other hand.

## Results:

The pretesting analysis delivered no evident disparities in clinical and demographic characteristics between the study and control groups. Mild dysphagia was also similarly distributed in both groups, at 90% in the experimental group and 93.33% within the control group. A majority of participants within the experimental group also described significant throat pain, 63.33%, as against 60% in the control group. The observed p-values of 0.57 for dysphagia and 0.64 for throat pain suggest no statistically significant differences between the groups studied. The findings indicate that both groups exhibited comparable postoperative symptoms regarding these specific issues. In post-test I, the experimental and control groups observed an essential difference in throat pain (p = 0.05) and dysphagia (p = 0.01). This difference was even more pronounced in post-test II, where throat pain (p = 0.001) and dysphagia (p = 0.001) demonstrated highly significant p-values, revealing even greater disparities between the groups (Table 1).

**Figure 1:**
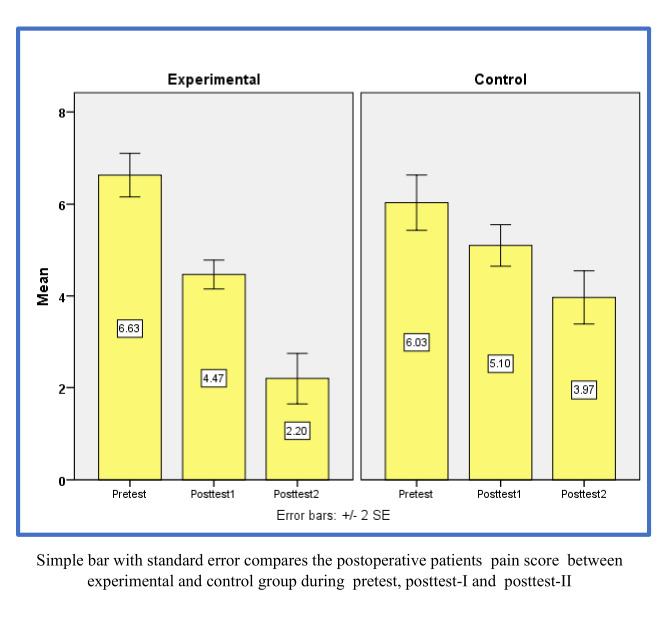
A simple bar with standard error contrasts the pain levels of postoperative patients in experimental and control groups during pre-test, post-test-I and post-test-II.

**Figure 2:**
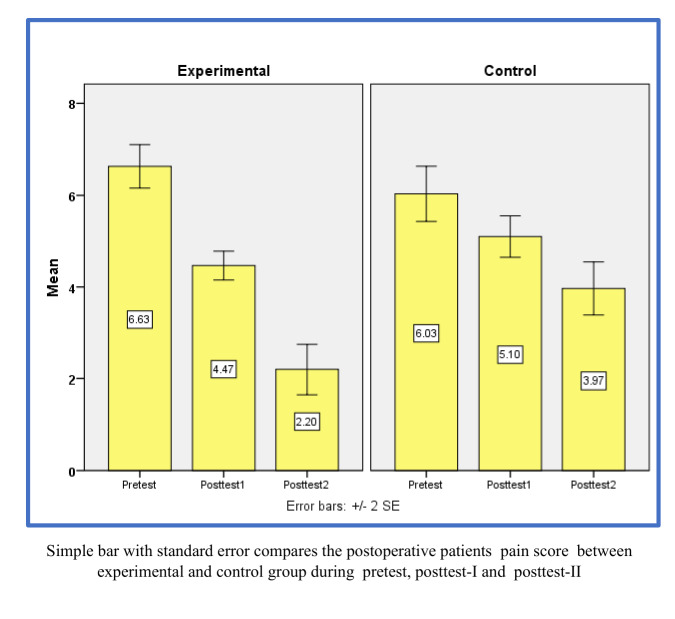
A simple bar with standard error is used to compare postoperative dysphagia scores in both experimental and control groups, pre-test and post-test-I and post-test-II.

The repeated measures ANOVA F-test analysis on the intervention group showed that the average total pain score significantly differed when comparing pre-test to post-test II, as shown in the table above (F = 178.89, P < 0.001) (Figure 1). Similarly, the control group also showed a significant difference in the mean overall pain scores between the pre-test and post-test II (F = 28.53, P < 0.001), based on an identical repeated measure F-test analysis. In the experimental cohort, patients undergoing post-operative assessment demonstrated, on average, a 44.30% decrease in pain scores in the post-test following the administration of the intervention. Conversely, in the control cohort, patients exhibited an average reduction of 20.60% in pain scores in the post-test after receiving standard care (Table 2). Comparing the pre-test and post-test-II dysphagia scores of the control group revealed a significant difference (F = 213.76, P < 0.001). Analysis of the repeated measures F-test also indicates that the mean scores for the control group between the pre-test and post-test-II are significantly different from each other (F = 66.39, P < 0.001) (Figure 2). The experimental cohort observed an average decrease of 21.58% in dysphagia scores post-test after the intervention compared to a control cohort, which only reduced by 9.66%. In post-test II, individuals in the experimental cohort aged less than 40 years, free of comorbidities and with a BMI score between 23 and 24.9 kg/m^2^ reported no pain. Chi-square analysis showed no association between the variables and pain levels in the control group. Moreover, the intervention group showed that patients performing semi-professional jobs, living in cities and showing higher independent activity levels did not experience dysphagia. On the other hand, the control group showed no significant associations between demographic or clinical characteristics and the post-test dysphagia scores. Both groups had been subjected to a chi-square test for the significance test.

## Discussion:

The initial levels of dysphagia and throat discomfort among post-operative patients within both the experimental and control cohorts is studied. The results indicated that 90% of participants in the experimental cohort and 93.33% of those in the control cohort experienced mild dysphagia, whereas 63.33% of the experimental cohort and 60% of the control cohort reported experiencing considerable throat pain. The p-values of 0.57 for throat pain and 0.64 for dysphagia in the statistical analysis reflected that the means did not differ significantly among the groups studied. Hence, both groups probably had comparable baseline conditions. The results of this study align with the investigation carried out by Yu *et al.* [7], which examined post-extubation dysphagia among 173 adult patients receiving intensive care. Their findings indicated dysphagia prevalence rates of 86.71%, 63.01% and 43.35% at the intervals of 1, 4 and 24 hours following extubation, respectively. The secondary aim was to assess post-test levels of both throat pain and dysphagia. In Post-Test I, 76.67% of the group showed mild throat pain as compared to 50% from the control group, while 53.33% of the experimental group revealed mild dysphagia in comparison to 83.33% of the control group. By Post-Test II, 60% of the experimental group had revealed no throat pain, while 80% of the control group still presented with mild discomfort. In addition, 80% of the experimental group had no dysphagia, while 63.33% of the control group continued to suffer from mild symptoms. The p-values were significantly high: 0.05 in Post-Test I for pain and 0.001 in Post-Test II and for dysphagia: 0.01 in Post-Test I and 0.001 in Post-Test II. Results in the present study have been derived from the research conducted by Eri *et al.* [8] to establish the effects of warm saline gargling post-extubation on sore throat. This study proved that demographic variations between groups were insignificant and the severity of the pain was reduced profoundly at different follow-up stages in the intervention group. The third goal was to evaluate decreased throat pain and irritation from the double moisture treatment method during swallowing. For the experimental group, repeated measure F-test results revealed significant differences between the patients' pre-test and Post-Test II means scores and it was reduced by 44.30% (F = 178.89, p ≤ 0.001). Variations were also found in the control group on the two measures, F = 28.53, p ≤ 0.001, with a decline of 20.60% in pain scores. Between the pre-test and Post-Test II drop for the experimental group, dysphagia had an F value of 213.76 with p ≤ 0.001 and a decline of 21.58%. Control has significant variations at an F of 66.39, p ≤ 0.001; these results revealed a 9.66% decline. Therefore, this evidence is valid for saying that the double moisture method does work and that its usage of H1 is deserved. Other adjunctive evidence emerged from reports by Arianto *et al.* [9], stating that magnesium sulfate gargle was more effective in relieving sore throat post-extubation than ketamine gargle. The fourth objective was the exploratory study regarding some clinical and demographic indicators associated with post-test scores of dysphagia and throat pain. Among the interventional group, less than 40 years old did not present with severe pain (χ^2^ = 16.56, p = 0.01), patients without comorbidity presented no pain (χ^2^ = 5.63, p = 0.05) and participants with a BMI of 23-24.9 kg/m^2^ presented no pain (χ^2^ = 6.67, p = 0.05). Control group: No significant relations were discovered. Dysphagia dimension: The test group demonstrated that semi-professionals χ^2^ = 9.53, p = 0.05, who reside in the town χ^2^ = 7.40, p = 0.01 and form of activity independently χ^2^ = 6.19, p = 0.05, had a low score on dysphagia and in the control group, no meaningful relations have been revealed. Therefore, H2 has been confirmed. This resonates with the findings of Epp *et al.* [10], who emphasized that considerable relationships exist between demographic factors and postoperative sore throat, explicitly noting that 'gender and intubation attempts impact post-operative outcomes.

## Conclusion:

Two-moisture therapy is yet another helpful non-pharmacological intervention that can reduce postoperative sore throat and dysphagia in surgical patients. The differences between the treatment group and the control were marked. This indicates that intervention produced some benefits in clinical outcomes. It would ultimately lead to recovery and increase patient satisfaction if included in the patient's treatment protocols. In the meantime, the constraints developed in the present study offer scope for further research to be validated and further continued.

## Figures and Tables

**Table 1:** Comparison of mean pain scores between experimental and control groups: pre-test, post-test I and post-test ii

**Group**	**Pre-test**		**Post-test-I**		**Post-test-II**		**(Pre-test, post-test-II) mean difference**	**One-way repeated measures ANOVA F-test**
	**Mean**	**SD**	**Mean**	**SD**	**Mean**	**SD**		
Experimental	6.63	1.3	4.47	0.9	2.2	1.5	4.43	F=178.89
								p=0.001*** (S)
Control	6.03	1.7	5.1	1.2	3.93	1.6	2.1	F=28.53
								p=0.001***
								(S)

**Table 2:** Comparative analysis of the average dysphagia scores of the experimental and control groups at pre-test, post-test i and post-test ii

**Group**	**Pre-test**		**post-test-I**		**post-test-II**		**(Pre-test, post-test-II) Mean difference**	**One-way Repeated measures ANOVA F-test**
	**Mean**	**SD**	**Mean**	**SD**	**Mean**	**SD**		
Experimental	12.93	2.9	7.1	3.4	4.3	3.1	8.63	F=213.76
								p=0.001*** (S)
Control	12.13	3.8	10.1	3.3	8.27	4.2	4.41	F=66.39
								p=0.001***
								(S)
